# Dual inhibition of histone deacetylases and phosphoinositide 3-kinase enhances therapeutic activity against B cell lymphoma

**DOI:** 10.18632/oncotarget.14876

**Published:** 2017-01-28

**Authors:** Patrizia Mondello, Enrico Derenzini, Zahra Asgari, John Philip, Elliott J. Brea, Venkatraman Seshan, Ronald C. Hendrickson, Elisa de Stanchina, David A. Scheinberg, Anas Younes

**Affiliations:** ^1^ Department of Medicine, Memorial Sloan Kettering Cancer Center, New York, NY, USA; ^2^ Proteomics Core Facility, Memorial Sloan Kettering Cancer Center, New York, NY, USA; ^3^ Molecular Pharmacology Program, Memorial Sloan Kettering Cancer Center, New York, NY, USA; ^4^ Department of Epidemiology and Biostatistics, Memorial Sloan Kettering Cancer Center, New York, NY, USA; ^5^ Antitumor Assessment Core, Memorial Sloan Kettering Cancer Center, New York, NY, USA

**Keywords:** MYC, PI3K, DLBCL, BCR pathway

## Abstract

Phosphoinositide 3-kinase (PI3K) and Myc are known to cooperate in promoting the survival and growth of a variety of B-cell lymphomas. While currently there are no small molecule inhibitors of Myc protein, histone deacetylase (HDAC) inhibitors have been shown to reduce levels of Myc protein by suppressing its transcription. We assessed the efficacy of CUDC-907, a new rationally designed dual inhibitor of PI3K and HDACs, in a panel of lymphoma cell lines. CUDC-907 treatment resulted in a dose- and time-dependent growth inhibition and cell death of DLBCL cell lines, irrespective of the cell of origin. CUDC-907 treatment down-regulated the phosphorylation of PI3K downstream targets, including AKT, PRAS40, S6, and 4EBP1, increased histone 3 acetylation, and decreased Myc protein levels. SILAC-based quantitative mass spectrometry demonstrated that CUDC-907 treatment decreased the protein levels of several components of the B cell receptor (BCR) and Toll like receptor (TLR) pathways, including BTK, SYK, and MyD88 proteins. These cellular changes were associated with an inhibition of NF-kB activation. CUDC-907 demonstrated *in vivo* efficacy with no significant toxicity in a human DLBCL xenograft mouse model. Collectively, these data provide a mechanistic rationale for evaluating CUDC-907 for the treatment of patients with Myc and PI3K-dependent lymphomas.

## INTRODUCTION

There is clear evidence for the oncogenic cooperation between Myc and activated phosphoinositide 3-kinase (PI3K) signaling pathway in lymphomagenesis, providing an opportunity for developing mechanism-based therapy to disrupt this cooperative survival mechanism. Constitutive expression of AKT accelerated lymphomagenesis and resistance to chemotherapy in Eμ-Myc mouse model [1]. Similarly, combining constitutive c-Myc expression with constitutive PI3K activity in mouse germinal center B cells resulted in Burkitt lymphoma-like tumors [2]. Furthermore, analysis of primary human Burkitt lymphoma (BL) tissue sections revealed that two-thirds of the cases expressed high levels of phosphorylated AKT and S6 proteins, indicative of PI3K and mTORC1 activation [2].

Gene expression profiling has distinguished mainly two distinct molecular cell-of-origin subtypes: activated B cell-like (ABC) and germinal center B cell-like (GCB) [3, 4]. Constitutive activation of the NF-kB pathway is the hallmark of ABC DLBCL [5, 6], while the PI3K pathway promotes cell proliferation in GCB DLBCL [6]. Recently, genetic and proteomics studies have identified a prognostic role for Myc and Bcl2 genetic translocations and/or protein co-expression [7]. Most published series reported about 10% of DLBCL harbored Myc, Bcl2 and/or Bcl6 translocations and predominantly seen in the subset of GCB subtype [7–13]. This category was called “double hit” lymphoma (DHL) or triple hit lymphoma and now is recognized as “high grade B-cell lymphoma (HGBL) with rearrangements of Myc and Bcl2 and/or Bcl6 [14]. Prior attempts to develop small molecule inhibitors that specifically and directly target c-Myc protein have been unsuccessful. However, Myc cellular protein abundance can be decreased by using epigenetic modifying drugs (HDAC inhibitors or bromodomain/BET inhibitors) that are known to inhibit Myc transcription [15, 16].

In this study, we investigated the ability of CUDC-907, a new chemically-designed, oral small molecule that combines the active hydroxamate moiety of HDAC inhibitor with a PI3K inhibitor into a single scaffold [17]. We demonstrate that CUDC-907, as a single agent, is capable of disrupting the oncogenic cooperation between Myc and PI3K, providing a rationale for evaluating this novel compound in patients with Myc and PI3K-dependent lymphomas.

## RESULTS

### *In vitro* activity of CUDC-907 in lymphoma cell lines

**Figure 1:**
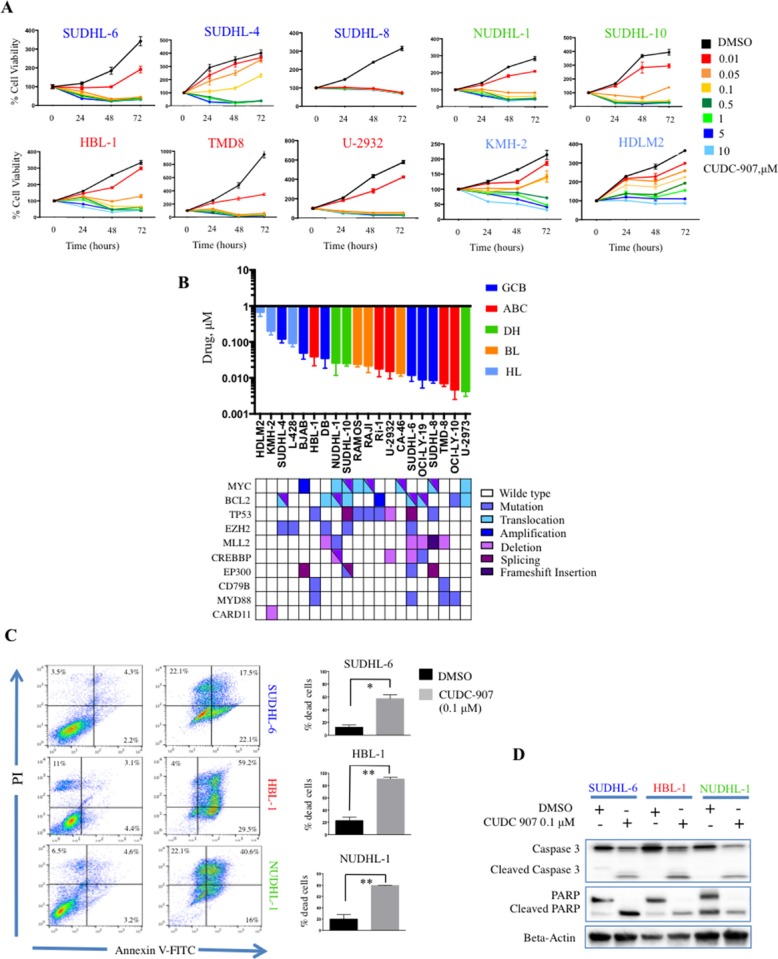
Antiproliferative activity of CUDC-907 in B-cell lymphoma cell lines (**A**) MTS assay of 8 representative DLBCL and 2 Hodgkin lymphoma cell lines treated with increasing dose of CUDC-907 from 0.01 to 10 μM for 24, 48, 72 hours. Error bars represent standard error of the mean (S.E.M) of triplicate experiments. (**B**) Bar graph showing IC 50 values of CUDC-907 in B cell (*n* = 17) and Hodgkin lymphoma (*n* = 3) cell lines after treatment for 72 hours (upper panel). CUDC-907 demonstrated efficacy irrespective of the cell of origin, genetic alterations or mutations of histone modifiers genes, Myc and BCL-2 rearrangements. Viability was determined by MTS assay. Error bars represent S.E.M. of triplicate experiments. (**C**) CUDC-907 induces apoptosis in lymphoma cell lines. SUDHL-6, HBL-1, NUDHL-1 and KMH-2 cells were treated for 24 hours with CUDC-907 0.1 μM before they were stained with propidium iodide and annexin V and analyzed by flow cytometry (left panel). Bar graphs summarizing the results of 3 independent experiments in SUDHL-6, HBL-1, NUDHL-1 and KMH-2 cells. Each bar represent the percentage of dead cells shown in the right upper and lower quadrants (annexin positive cells). Error bars represent S.E.M. of triplicate experiments. Differences between groups were calculated with the Student's t test. *p < 0.05; **p < 0.005. (**D**) Representative western blot showing caspase 3 cleavage and PARP cleavage after 24 hours of incubation with 0.1 μM CUDC-907 in SUDHL-6, HBL-1 and NUDHL-1 cells lines, but not in KMH-2 cells.

To assess the effect of CUDC-907 on cell proliferation, cells were incubated with increasing drug concentrations (from 0.01 to 10 μM) for 24, 48 and 72 hours (hrs). CUDC-907 treatment resulted in growth inhibition in a dose and time dependent manner (Figure 1A) with an IC50 < 0.1 μM in 17 out 20 (82%) lymphoma cell lines at 72 hrs (Figure 1B) (Supplementary Table 1). CUDC-907 demonstrated activity in both ABC and GCB) [4] cell lines irrespective of genetic alterations, including the presence of dual translocation involving c-Myc and Bcl2 (DHL) (Figure 1B). Using Annexin V- propidium iodide staining, we found that CUDC-907 induced cell death by apoptosis after 24 hrs at low concentration (0.1 μM) in three representative DLBCL cell lines, SUDHL-6 (GCB), HBL-1 (ABC) and NUDHL-1 (DHL), but was ineffective in the Hodgkin lymphoma (HL) cell line KMH-2 (Figure 1C). Consistent with these data, the induction of apoptosis was associated with caspase 3 and PARP cleavage in the sensitive DLBCL cell lines, but not in the HL cell line (Figure 1D).

### CUDC-907 downregulates c-Myc and PI3K downstream targets

**Figure 2:**
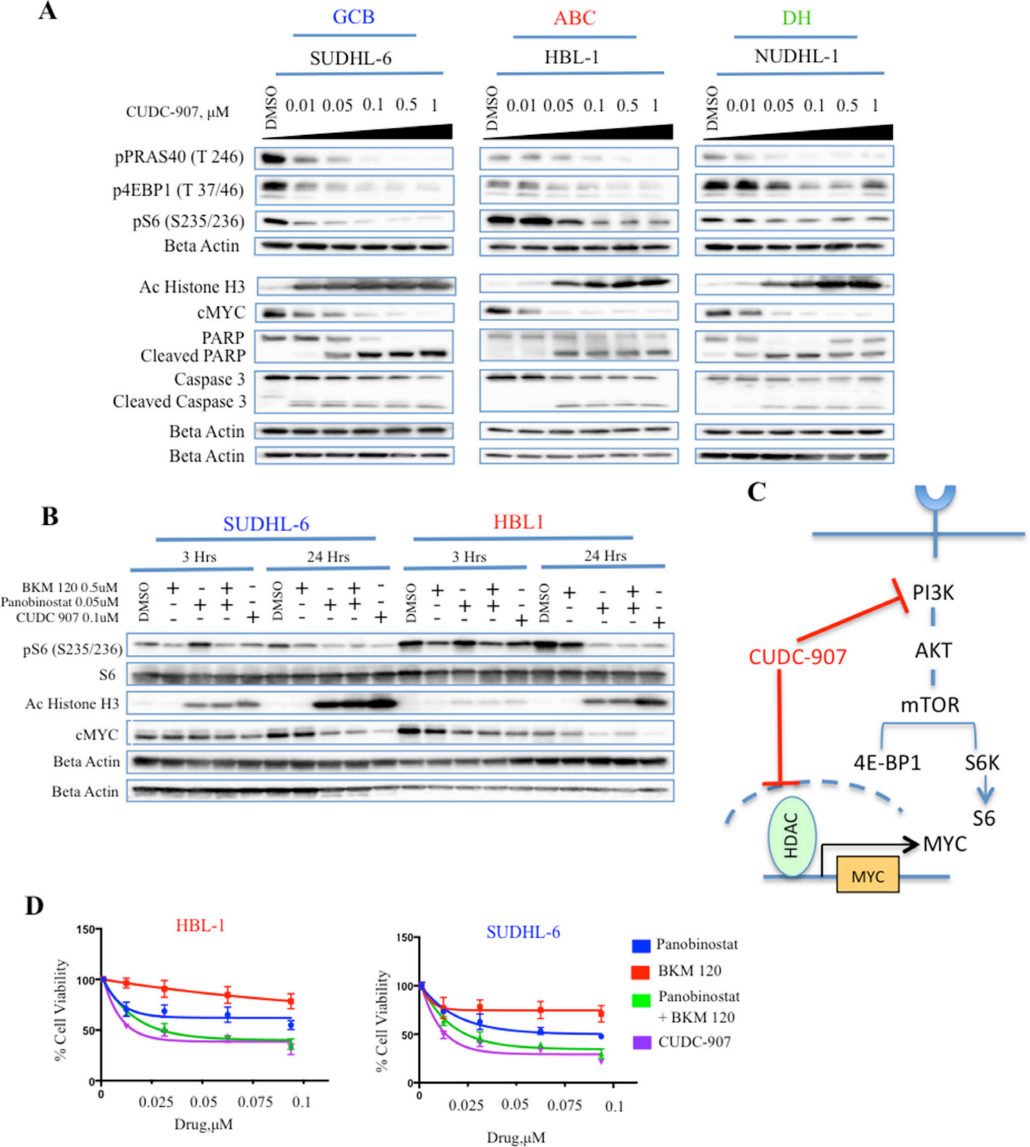
CUDC-907 inhibits c-Myc and HDACs in DLBCL cell lines (**A**) Western blot analysis demonstrating the effect of increasing concentration of CUDC-907 (from 0.01 to 1 μM) on PI3K downstream targets (p4EBP1, pPRAS40 and pS6) and histone H3 acetylation (Ac Histone H3). The actual inhibition of PI3K and HDAC resulted in a decrease in c-Myc protein levels. Activation of caspase 3 and PARP was present as well. (**B**) Western blot analysis showing the effect of single agent PI3K inhibitor (BKM-120), HDAC inhibitor (panobinostat), or both on PI3K and HDAC targets. The data show that CUDC-907 produces similar results to the combination of BKM-120 and panobinostat. (**C**) CUDC-907 combines PI3K and HDAC inhibitor into a single scaffold, and therefore is capable of disrupting the oncogenic cooperation between PI3K and c-Myc. (**D**) MTS assay demonstrating that CUDC-907 inhibits cell proliferation, mimicking the effect of BKM-120 plus panobinostat. Cells were incubated with increasing concentrations of BKM-120, panobinostat and CUDC-907, and viability assessed after 24 hours.

To investigate the mechanism of action of CUDC-907 we first examined its effect on PI3K and HDAC targets. As expected, CUDC-907′s inhibition of HDAC resulted in an increase of acetylated histone 3, leading to a decrease of c-Myc protein levels (Figure 2A). Similarly, CUDC-907 inhibited PI3K pathway activation, as indicated by the dose-dependent decreases in phosphorylation of downstream targets (p4EBP1, pPRAS40 and pS6) in the sensitive B-cell lines (Figure 2A). Using a multiplex assay for pAKT (Thr 308) we observed inhibition at 30 minutes. However, AKT phosphorylation progressively increased in most cells in time dependent manner (Supplementary Figure 1) likely due to the loss of the negative feedback loop between pS6 and insulin receptor substrate 1 (IRS-1) [18].

Next, we investigated the molecular mechanism of each single agent using the HDAC inhibitor panobinostat and the PI3K inhibitor BKM-120, alone and in combination at their IC50 concentrations (Supplementary Table 2), and compared the results to single agent CUDC-907. As shown in Figure 2B and 2C, treatment with single agent CUDC-907 produced similar molecular effects on pS6, acetylated H3, and c-Myc levels compared with the combination of panobinostat and BKM-120. These molecular changes were associated with similar biologic antiproliferative activity in DLBCL cell lines (Figure 2D). Collectively, these data demonstrate that CUDC-907 is indeed a dual inhibitor of PI3K and HDACs, with antiproliferative activity similar to the combination of BKM-120 and panobinostat.

### Quantitative proteomics identifies novel targets of CUDC-907 in DLBCL

**Figure 3:**
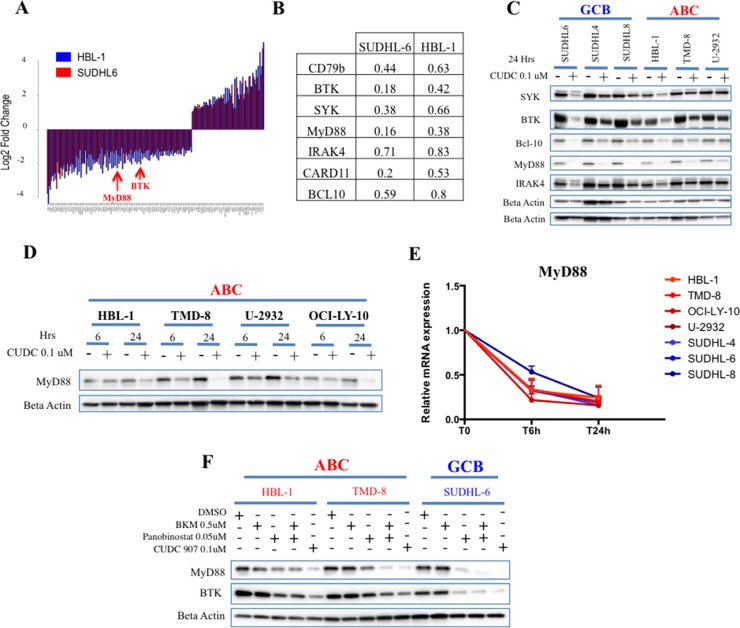
Quantitative proteomics identifies B-cell receptor signaling proteins as targets for CUDC-907 in DLBCL cell lines (**A**) Summary graph showing fold change cellular proteins that are commonly up or down-regulated in both HBL-1 (blue) and SUDHL-6 (red) cells. (**B**) A subset analysis of the experiments shown in Figure 3A, demonstrating the effect of CUDC-907 treatment (0.1 μM for 24 hours) on proteins involved in BCR and TLR signaling. Each value represents the average of the fold change (CUDC-907 vs DMSO) obtained in the forward and the reverse experiments. (**C**) Western blot confirming the effect of CUDC-907 (0.1 μM for 24 hours) on BCR and TLR signaling proteins in B-cell lymphoma cell lines (GCB n = 3 and ABC n = 3). (**D**) Western blot demonstrating the effect of CUDC-907 (0.1 μM) on MyD88 protein in a panel of ABC DLBCL cells (HBL-1, TMD-8 and OCI-LY-10 harbor the MyD88 mutation L265P). (**E**) Change in relative mRNA levels of MyD88 over time in B-cell lymphoma cell line panel (GCB n = 3 and ABC = 4) after treatment with CUDC-907 0.1 μM for the indicated time. (**F**) Western blot demonstrating that CUDC-907 or panobinostat can decrease cellular abundance of MyD88 and BTK in DLBCL cell lines. The analysis was performed after 24 hours of incubation with each drug at the indicated concentration.

To identify additional downstream targets of CUDC-907, we performed a stable isotope labeling of amino acids in cell culture (SILAC)–based quantitative mass spectrometry. We applied this technique to two representative DLBCL cell lines treated with 0.1 μM CUDC-907 for 24 hrs (HBL-1 of ABC origin, and SUDHL-6 of GCB origin). Of the 5447 proteins detected in both HBL-1 and SUDHL-6 cells, 120 proteins were commonly increased or decreased after treatment with CUDC-907 in both cell lines (Figure 3A and Supplementary Table 3). CUDC-907 consistently decreased the level of proteins involved in B cell receptor (BCR) and Toll like receptor (TLR) signaling (Figure 3B and Supplementary Figure 2B). These effects were confirmed in a wider range of DLBCL cell lines by western blots (Figure 3C). Because the gain-of-function mutation MyD88 L265P is well known to confer oncogenic properties in DLBCL [3, 19], we further examined the effect of CUDC-907 in cell lines that harbor this mutation. As shown in Figure 3D, CUDC-907 reduced MyD88 protein level in all the mutant cell lines. Furthermore, the reduction in MyD88 protein level was associated with a rapid and profound decrease in MyD88 mRNA level irrespective of the mutation status (Figure 3E). The effect of CUDC-907 on MyD88 protein was predominantly driven by its HADC inhibitory property, as similar effect was observed by treating the cells with panobinostat, but not with the PI3K inhibitor BKM-120 (Figure 3F).

**Figure 4:**
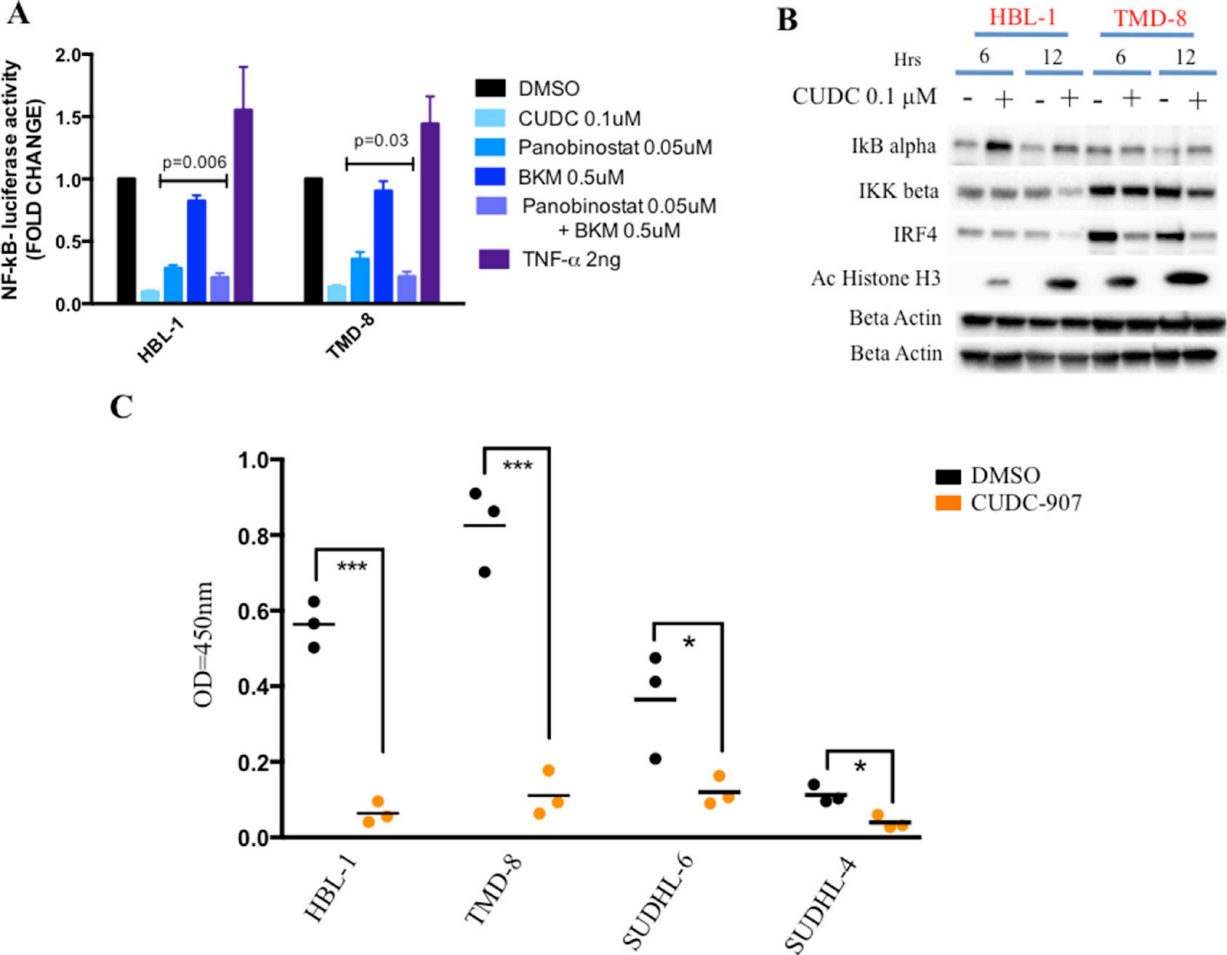
CUDC-907 inhibits NF-kB signaling in ABC DLBCLs (**A**) Relative NF-kB-luciferase activity in two representative ABC DLBCL cell lines (HBL-1 and TMD-8). Cells were treated for 12 hours with indicated concentration of either panobinostat, BKM-120, the combination of these two drugs, CUDC-907 or DMSO. Cells were incubated with 2 ng of TNF-α as positive control for NF-kB activation. Error bars represent standard error of the mean (S.E.M) of triplicate experiments. (**B**) Western blot demonstrating that CUDC-907 (0.1 μM for 6 and 12 hours) inhibits NF-kB activation by increasing IkB alpha levels and decreasing IKK beta levels in two representative ABC DLBCL cell lines (HBL-1 and TMD-8). The changes were associated with a decrease in IRF4 levels (see Supplementary Figure 2B). (**C**) ELISA assay showing a more pronounced decrease of nuclear NF-kB p65 in two representative ABC (HBL-1 and TMD-8) cell lines compared with two GCB (SUDHL-6 and SUDHL-4) treated with either 0.1 μM CUDC-907 or DMSO for 12 hours. Differences between groups were calculated with the Student's t test. *p < 0.05; ***p < 0.001.

Given that BCR and TLR signaling leads to NF-kB activation (Supplementary Figure 2C), we investigated whether CUDC-907 treatment can also inhibit NF-kB. Therefore, we treated two representative ABC cell lines, which are characterized by aberrant activation of NF-kB [5, 6], with CUDC-907 for 12 hrs and determined its effect on NF-kB activity using a luciferase promoter assay containing NF-kB binding site motif. As shown in Figure 4A, CUDC-907 inhibited NF-kB activity in both cell lines, an effect that was reproduces by panobinostat but not by BKM-120. CUDC-907 treatment was associated with up-regulation of inhibitor of kappa B alpha (IkBα) and down-regulation of inhibitor of NF-kB subunit beta (IKK beta) and IRF4 consistent with NF-kB pathway inhibition (Figure 4B and Supplementary Figure 3). CUDC-907 treatment (0.1 μM CUDC-907 for 12 hours) resulted in a marked reduction in NF-kB nuclear activity, which was more pronounced in the ABC cell lines (HBL-1 and TMD-8) compared with the GCB cell lines (SUDHL-6 and SUDHL-4) (Figure 4C).

### *In vivo* efficacy of CUDC-907 in DLBCL xenograft model

**Figure 5:**
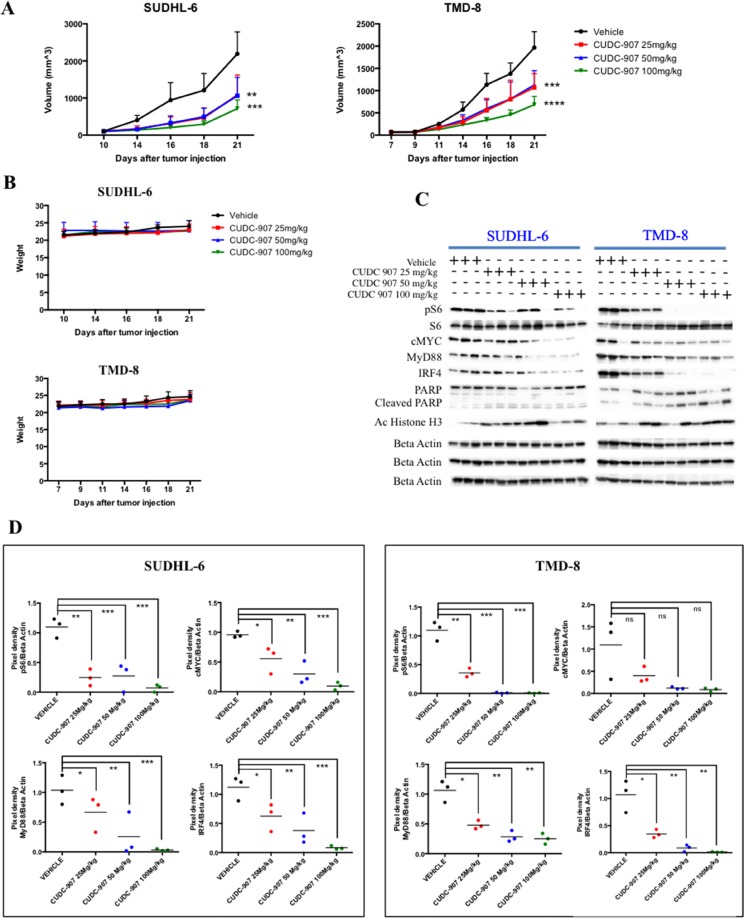
CUDC-907 therapy in xenograft model of GCB and ABC DLBCL (**A**) Human SUDHL-6 and TMD-8 DLBCL cells were established as s.c. tumors in NOD/SCID mice and treated orally with vehicle (*n* = 8) or CUDC-907 at 25 (n = 8), 50 (*n* = 8) and 100 (*n* = 8) mg/kg, 5 times weekly. CUDC-907 inhibits tumor growth in a dose-dependent manner when compared with the control group. Tumor volume was measured 3 times per week. Differences between groups were calculated with the Student's t test. **p < 0.005; ***p = 0.0001; ****p < 0.00001. (**B**) Variations of body weight over time in SUDHL-6 and TMD-8 xenografts treated with CUDC-907 at 25, 50 and 50 mg/kg. (**C**) In vivo effects of CUDC-907 on pS6, c-Myc, MyD88, IRF4, PARP and acetyl histone H3 levels in SUDHL-6 and TMD-8 xenografts after 24 hours from the last drug administration. Proteins were extracted from tumor tissues and analyzed by western immunoblotting to assess in vivo inhibition of indicated targets. (**D**) Scatterplots summarizing changes in expression levels of pS6, c-Myc, MyD88 and IRF4, expressed as ratios of pixel densities between actin and protein of interest. Densitometry was performed by using the ImageJ software. Differences between groups were calculated with the Wilcoxon Rank test. *p < 0.05; **p < 0.01; ***p < 0.001; ns, not significant.

We next investigated the efficacy of CUDC-907 in a xenograft model of GCB and ABC DLBCL. NOD. Cg-Prkdc*^scid^* Il2rg*^tm1Wjl^*/SzJ (NSG) mice implanted subcutaneously with SUDHL-6 and TMD-8 cell-derived tumors were treated orally with either vehicle or CUDC-907 at 25, 50 and 100 mg/kg, 5 times weekly [17]. CUDC-907 produced a significant inhibition of DLBCL tumor growth in a dose-dependent manner. The most growth delay was observed at 100 mg/kg in both models (Figure 5A). Overall, CUDC-907 was well tolerated, as it did not induce a significant weight loss, or other clinical signs, in treated mice (Figure 5B). We evaluated also the *in vivo* activity of CUDC-907 on its molecular targets in harvested tumors by western blot. CUDC-907 therapy resulted in down-regulation of PI3K and NF-kB signaling pathways as indicated by decrease in pS6 and IRF4 24 hrs post dosing (Figure 5C, 5D). Similarly, CUDC-907 inhibited HDACs leading to an increase in histone H3 acetylation and a decrease in c-Myc and MyD88 protein levels (Figure 5C, 5D). Relatively increased of PARP was also detected as marker of apoptosis (Figure 5C).

## DISCUSSION

Several previous studies demonstrated the oncogenic cooperation between Myc and PI3K signaling pathway in a variety of lymphomas [1, 2]. As HDAC inhibitors can inhibit Myc transcription [15, 16], several investigators attempted disrupting Myc and PI3K cooperation by combining HDAC inhibitors and PI3K pathway inhibitors [20, 21]. A synergic activity was demonstrated in DLBCL irrespective of subtype [20]. In this study, we demonstrate that CUDC-907, as a single agent, can reproduce the molecular and biologic activity of the two drugs in combination (HDAC inhibitor plus PI3K inhibitor). At the molecular level, CUDC-907 inhibited the phosphorylation of PI3K downstream targets, in addition to inhibition of HDACs, resulting in an increase in histone H3 acetylation and Myc down-regulation (Figure 2C). As a consequence, CUDC-907 induced caspase activation, PARP cleavage, and cell death in different subtypes of DLBCL cell lines, but not in the less-sensitive Hodgkin lymphoma cells. Furthermore, CUDC-907 decreased the cellular abundance of key proteins involved in BCR and TLR signaling pathways, resulting in inhibition of NF-kB activation. The *in vitro* activity was confirmed in a human DLCBL xenograft model, whereas CUDC-907 was capable of inhibiting PI3K and HDACs *in vivo*. In a previous study CUDC-907 has been shown to be more effective than PI3Ki and HDACi in a B lymphoma mouse model [17]. We demonstrated that CUDC-907 induced growth inhibition of both GCB and ABC DLBCL subtypes. These findings indicate that CUDC-907 may have a role in the treatment of Myc-expressing DLBCL and ABC-DLBCL, which are known to have a poor outcome when treated with standard combination chemotherapy [22].

Our preclinical observations provided a rationale for evaluating CUDC-907 in patients with relapsed DLBCL, especially those with high Myc expression. Results from a recently published phase-I study demonstrated the safety and clinical efficacy of CUDC-907 in patients with relapsed DLBCL [23]. A major advantage of combining HDAC and PI3K inhibitors in a single scaffold is the simplicity and ease of administration, likely resulting in improved patients compliance. Furthermore, combining both drugs in a single scaffold simplifies future combination strategies, especially with other novel small molecule inhibitors. However, because each CUDC-907 molecule combines an HDAC inhibitor and a PI3K inhibitor in a 1:1 fixed ratio, it is not possible to adjust the dose of each component separately to improve treatment efficacy or to decrease drug-related toxicity.

The ability of CUDC-907 to modulate BCR and TLR signaling may indicate that the drug should be examined in combination with BCR signaling inhibitors, such as SYK or BTK inhibitors. Furthermore, because CUDC-907 down-regulated the expression of MyD88 transcripts and protein, irrespective of its mutation status, future studies could focus on investigating a potential role for CUDC-907 in overcoming resistance to ibrutinib in DLBCL carrying MyD88 mutations.

## MATERIALS AND METHODS

### Cell lines

The human diffuse large B-cell lymphoma (DLBCL)-derived cell lines SUDHL-4, SUDHL-6, OCI-LY-19, U-2932, NUDHL-1, OCI-LY-3, Ri-1 and the Hodgkin cell lines HDLM2, KMH-2 and L-428 were obtained from the German Collection of Microorganisms and Cell Cultures, Department of Human and Animal Cell Cultures (Braunschweig, Germany); DB, SUDHL-8, SUDHL-10 and the BL cell lines RAJI, RAMOS and CA-46 were obtained from ATCC. The DLBCL derived cell lines (HBL-1, TMD-8, BJAB) were provided by Dr. R.E. Davis (Houston, Tx). The cell lines OCI-LY-10 was provided by Dr. E Cesarman (New York, NY) (Supplementary Table 4). All cell lines were grown to log phase at 37°C, in the presence of 5% CO2 and cultured in RPMI 1640 medium supplemented with 10 to 20% heat-inactivated fetal bovine serum (Hyclone, GE Healthcare Life Sciences), 1% L-glutamine, and penicillin-streptomycin in a humid environment of 5% CO2 at 37°C. OCI-LY-10 was cultured in Hyclone Iscove's Modified Dulbecco's Medium (GE Healthcare Life Sciences) with 20% heat-inactivated fetal bovine serum (Hyclone, GE Healthcare Life Sciences), 1% penicillin-streptomycin, 0.1% 2-Mercaptoethanol (GIBCO BRL, Gaithersburg, MD). All cell lines were authenticated by MSKCC Genomic Core Facility using targeted deep sequencing assay of 585 cancer genes (HemePACT). Barcoded pools were sequenced on Illumina HiSeq 2500 to 500–1000× coverage per sample. Sequencing was compared to pooled normal tissue for control. We excluded mutations present in two databases of inherited variants (DBSNP and 1000 genomes) and included mutations found in COSMIC. Mutations absent in the databases of inherited variant and COSMIC but found in our human lymphoma database were included.

### Reagents

CUDC-907 was provided by Curis (Inc., Massachusetts). Panobinostat and BKM-120 were purchased from Selleckem (Houston, TX).

### *In vitro* proliferation assay

Cells were seeded in 96-well plates at 50,000 cell/100 μl/well with either vehicle (DMSO 0.1%) or increasing concentrations of drugs for 24 to 72 hours. Cell viability was assessed with the non-radiactive cell proliferation MTS assay, using CellTiter96^©^ Aqueous One Solution Reagent (Promega, Madison, WI). The MTS reagent was added to the culture medium at 1:5 dilution, according to manufacturer`s instructions. Procedures to determine the effects of certain conditions on cell proliferation and apoptosis, were performed in 3 independent experiments. The 2-tailed Student *t* test and Wilcoxon Rank test were used to estimate the statistical significance of differences between results from the 3 experiments. Significance was set at *P* < .05. The PRISM software was used for the statistical analyses.

### Western blotting

Cells were pelleted by centrifugation, washed once with ice-cold PBS, and lysed on ice for 30 min using the Cell Signaling lysis buffer (#9803) according to manufacturer`s extraction protocol. Protein quantitation was done using the Direct Detect system (Millipore). A total of 30 μg of protein was denatured in Laemli buffer at 95°C for 5 minutes and western immunoblotting was performed using the Bio-rad system (TGX 4–15% gels). Transfer was performed using the Trans Blot turbo system (Bio-rad) into PVDF membranes. Images were acquired by using the Bio-rad Imaging Chemidoc MP system. Secondary anti-rabbit and anti-mouse HRP-conjugated antibodies were purchased from Bio-rad (#170-6515, #170-6516). Proteins were detected using the following antibodies purchased from Cell Signaling Technology: Acetyl Histone H3 Lys9 (#9649), Caspase3 (#9668), PARP (#9542), pPRAS40 (#13175), p4EBP1 (#2855), pS6 (#4856), S6 (#2217), BTK (#3533), SYK (#13198), MyD88 (#4283), BCL10 (#4237), IRAK4 (#4363), IkBa (#4812), IKKb (#2370). c-MYC (#32072) was purchased from Abcam. Beta-Actin was from SIGMA (A5316#).

### PCR pathway arrays and qPCR

Total RNA was extracted with the Qiagen (Valencia, CA) RNeasy mini kit protocol. A total of 1 μg of RNA was converted to cDNA using iScript cDNA synthesis kit (Bio-rad). Real-time polymerase chain reaction (PCR) was performed using the model CFX96 (Bio-rad). Primers for MyD88 and GAPDH were purchased from Biorad: MyD88 (qHsaCED0046947), and GAPDH (qHsaCED0038674).

### Flow cytometry - apoptosis

To detect apoptosis, annexin V conjugated with the fluorescent dye fluorescein isothiocyanate (FITC) (BD Pharmingen, #556547) was used. In short, cells were stained with annexin V and propidium iodide (PI) for 30 minutes at room temperature in dark. Cells were kept on ice before analysis by a flow cytometer (FACSCalibur; BD Biosciences).

### NF-κB reporter assays

To generate the nuclear factor kappa B (NF-kB) transcriptional reporters, HBL-1 and TMD-8 cell were first infected with a CMV Renilla luciferase lentivirus, to be used as internal control for normalization (QIAGEN). After hygromycin selection, cells were infected with a NF-κB reporter lentivirus, where the expression of firefly luciferase is under control of a synthetic minimal (m)CMV promoter and tandem repeats of the NF-kB transcriptional response element (TRE) (QIAGEN), and selected with puromycin. For drug experiments, cells were counted and seeded in fresh media at 1 × 10^6^ cells per mL before treatment for the indicated times. Luciferase activity was measured in 96-well plates, by using the Dual-Luciferase Reporter Assay System (Promega) according to manufacturer's instruction. Readings were performed on a Microtiter Plate Luminometer (PerkinElmer).

### Mass spectrometry studies

Cell culture, lysis and insolution digestion. Cells were grown as suspension cultures in RPMI media supplemented with 10% FBS and penicillin and streptomycin either unlabeled L-arginine (Arg0) and L-lysine (Lys0) at 50 mg/liter or equimolar amounts of the isotopic variants [U-13C6]-L- arginine (Arg10) and [U-13C6]-L–lysine HCl L-lysine (Lys6), (Cambridge Isotope Laboratories). After five cell doublings in suspension flask, cells were > 99% labeled with the isotopes. Cells were collected, washed with ice cold PBS and frozen in liquid nitrogen. Cells were thawed on ice and lysed in in RIPA buffer and mixed 1:1 based on protein quantity by micro BCA (Thermo Fisher), separated by SDS/PAGE, and stained with Simply Blue Stain Reagent (Life Technologies); and 15 gel sections excised with *in situ* trypsin digestion of polypeptides in each gel slice was performed as described [24]. The tryptic peptides were desalted by using a 2-μL bed volume of Poros 50 R2 reversed-phase beads (Applied Biosystems) packed in Eppendorf gel-loading tips [25]. The purified peptides were diluted to 0.1% formic acid, and each gel section was analyzed separately by microcapillary LC with tandem MS by using the NanoAcquity system (Waters) with a 100-μm inner diameter × 10-cm length C18 column (1.7 μm BEH130; Waters) configured with a 180-μm × 2-cm trap column coupled to an OrbiElite mass spectrometer (Thermo Fisher Scientific). Peptides were eluted with a linear gradient of 0–50% acetonitrile (0.1% formic acid) in water (0.1% formic acid) over 200 mins with a flow rate of 250 nL/min. Key parameters for the mass spectrometer were: automatic gain control (AGC) 3 × 10^6^ ions, resolution 70,000, top 10 DDA method, min signal 1 × 10^5^ with CID MS/MS collected in the ITMS. Tandem MS fragmentation spectra were searched for protein identification by using the Andromeda search engine (maxquant.org) against the reversed and concatenated Uniprot human protein database (downloaded from Uniprot Jan 24, 2014). One unique peptide was required for high-confidence protein identifications, and a minimum ratio count of two peptides (one unique and one razor) was required for SILAC ratio determination. Normalized SILAC ratios (i.e., H/L) were used for subsequent analysis. All MS/MS samples were analyzed by using MaxQuant (Max Planck Institute of Biochemistry; version 1.5.1.0) at default settings with a few modifications [26]. The default was used for first search tolerance and main search tolerance: 20 ppm and 6 ppm, respectively. Labels were set to Arg(10) and Lys(6) and enzyme specificity set as trypsin digestion with as many as two missed cleavages. Peptide, site, and protein FDR were all set to 1% with a minimum of one peptide needed for identification but two peptides needed to calculate a protein level ratio. The following modifications were used as variable modifications for identifications and included for protein quantification: oxidation of methionine, acetylation of the protein N terminus, and phosphorylation of serine, threonine, and tyrosine residues and carbamidomethyl modification on cysteine as a fixed mod. Raw data as well as original MaxQuant result files can be provided upon request.

### Mass spectrometry data analysis

5443 proteins were initially identified. Proteins with a missing value in one of the 2 cell lines or both were removed, as were proteins where one replicate fold change value was 10 times the other. 4027 proteins were then evaluable for the final analysis. Only proteins changing by at least 1.5 fold change in both forward and reverse experiments were considered as significantly deregulated.

### ELISA

Standard ELISA was performed using the nfKb P65 Transcription Factor Assay kit from Abcam (Cambrifge, MA) (#ab13312) according to manufacturer`s instructions.

### Xenograft studies

NSG mice (Jackson Laboratories) were used for *in vivo* on protocol approved by the Memorial Sloan Kettering Cancer Center Institutional Animal Care and Use Committee. Six week old female mice were injected subcutaneously with either 10 million TMD8 cells or 10 million SUDHL6 cells together with matrigel. Once tumors reached an average volume of 100 mm^3^, mice were randomized to receive either vehicle control or CUDC-907 at doses of 25 mg/kg, 50 mg/kg or 100 mg/kg p.o. daily, 5 times weekly for up to 3 weeks. Mice were observed daily throughout the treatment period for signs of morbidity/mortality. Tumors were measured three times per week using calipers, and volume was calculated using the formula: length × width2 × 0.52. Body weight was also assessed twice weekly. 8 hours and 24 hours after the last treatment, mice were sacrificed and tumors collected for immunoblot.

### Statistical analysis

Statistical significance was determined by Student *t* test. *p* values < .05 were considered significant (*p < 0.05; **p < 0.001; ***p < 0.001; ****p < 0.0001). Survival was estimated with the Kaplan-Meier survival curve method and differences in survival were calculated by long-rank test (Graph Pad Prism 6.0).

## SUPPLEMENTARY MATERIALS FIGURES AND TABLES




