# Supplementary material for: Dual inhibition of histone deacetylases and phosphoinositide 3-kinase enhances therapeutic activity against B cell lymphoma
Source: Oncotarget. 2017 Jan 28;8(8):14017–28. doi: 10.18632/oncotarget.14876 (PMC5355158; doi:10.18632/oncotarget.14876)
Supplement: Supplementary file 1 [file oncotarget-08-14017-s001.pdf]

Dual inhibition of histone deacetylases and phosphoinositide 3-kinase enhances therapeutic activity against B cell lymphoma

Supplementary Materials

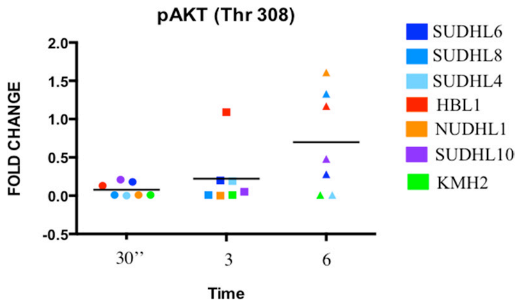

Supplementary Figure 1: Scatterplot summarizing the results of phospho AKT (Thr 308) assessed by luminex multiplex assay, showing significant decrease in phosphorylation levels at 30 minutes in our cell line panel (*n* = 7) after treatment with CUDC-907 0.1 µM for 24 hours. However, AKT phosphorylation progressively increased in most cells in time dependent manner.

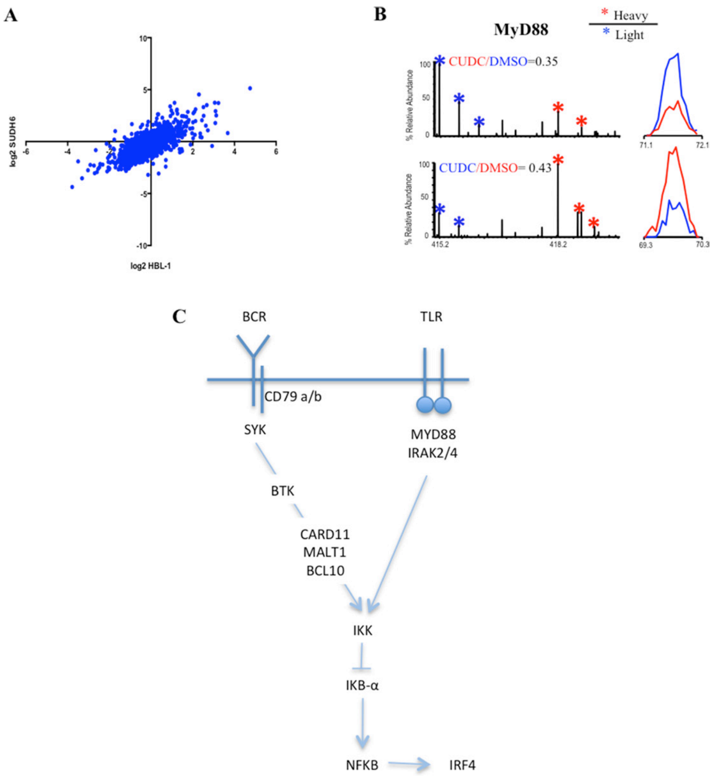

Supplementary Figure 2: (**A**) Scatterplot of quantitative proteomics data showing protein changes in HBL-1 (x axis) and SUDHL-6 cells (y axis) in a log2 scale. Average log2 fold change of forward and reverse experiments of HBL-1 and SUDHL-6 cells were used to generate the scatterplot. (**B**) Representative MS spectra from MyD88 are shown with forward (top panel) and reverse (lower panel) experiments after treatment with CUDC-907 0.1 µM for 24 hours in HBL-1. The selected ion current chromatograms for the (M+2H)2+ ions from tryptic peptide LLELLTK for the forward and reverse experiments are shown to the right of the associated spectrum. The light isoform (m/z = 415.3) is shown in blue and heavy isoform (m/z = 418.3) is shown in red. The integrated area under the curve was used to calculate the ratio of the two isoforms. (**C**) Schematic representation of the B-cell receptor (BCR) and Toll like receptor (TLR) pathways, which converge to activate NF-kB.

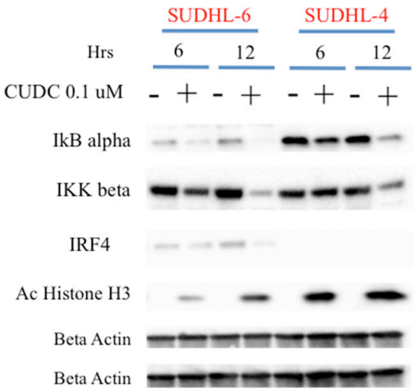

Supplementary Figure 3: Western blot demonstrating that CUDC-907 (0.1 µM for 6 and 12 hours) inhibits NF-kB activation by increasing IkB alpha levels and decreasing IKK beta levels in two representative GCB DLBCL cell lines (SUDHL-6 and SUDHL-4). The changes were associated with a decrease in IRF4 levels.

Supplementary Table 1: Table summarizing the CUDC-907 IC50 dose at 72 hours for all the lymphoma cell lines used

Cell line	CUDC-907 IC50 (µM) 72Hrs
**HDLM2**	0.6843
**KMH2**	0.2023
**SUDHL-4**	0.1224
**L-428**	0.0919
**BJAB**	0.0498
**HBL-1**	0.0394
**DB**	0.0355
**NUDHL-1**	0.0262
**SUDHL-10**	0.0254
**RAMOS**	0.0243
**RAJI**	0.0218
**Ri-1**	0.0182
**U-2932**	0.0153
**CA-46**	0.0135
**SUDHL-6**	0.0119
**OCI-LY-19**	0.0089
**SUDHL-8**	0.0087
**TMD-8**	0.0070
**OCI-LY-10**	0.0047
**U-2973**	0.0042

Supplementary Table 2: Table summarizing the panobinostat and BKM-120 IC50 dose at 48 hours for DLBCL cells

Cell line	Panobinostat IC50 (µM) 48 Hrs	BKM-120 IC50 (µM) 48 Hrs
**HBL-1**	0.033	0.65
**TMD-8**	0.085	0.9
**U-2932**	0.62	1.2
**SUDHL-8**	0.12	0.5
**SUDHL-6**	0.05	0.8
**SUDHL-4**	0.025	1.5
**OCI-LY-19**	0.01	0.1
**NUDHL-1**	0.007	0.5
**SUDHL-10**	0.05	0.8
**U-2973**	0.40	0.4

Supplementary Table 3: Proteins that are commonly down or up-regulated following treatment of HBL-1 and SUDHL-6 cells with 0.1 µM CUDC-907 for 24 hours. See_Supplementary_Table 3

Supplementary Table 4: Table summarizing the cell lines used including cell of origin and source

Cell line	Cell of Origin	Source
**Diffuse Large B cell Lymphoma**		
**SUDHL-4**	GCB	DSMZ
**SUDHL-6**	GCB	DSMZ
**OCI-LY-19**	GCB	DSMZ
**SUDHL-8**	GCB	ATCC
**BJAB**	GCB	MDCC (Davis lab)
**U-2932**	ABC	DSMZ
**OCI-LY-3**	ABC	DSMZ
**Ri-1**	ABC	DSMZ
**HBL-1**	ABC	MDCC (Davis lab)
**TMD-8**	ABC	MDCC (Davis lab)
**OCI-LY-10**	ABC	WCMC (Cesarman lab)
**NUDHL-1**	DH	DSMZ
**SUDHL-10**	DH	ATCC
**Hodgkin Lymphoma**		
**HDLM2**	-	DSMZ
**KMH-2**	-	DSMZ
**L-428**	-	DSMZ
**Large Cell Lymphoma B-Lymphoblast**		
**DB**	-	ATCC
**Burkitt Lymphoma**		
**RAJI**	-	ATCC
**RAMOS**	-	ATCC
**CA-46**	-	ATCC
